# Biomarkers of Oxidative Stress and Inflammation in Chronic Airway Diseases

**DOI:** 10.3390/ijms21124339

**Published:** 2020-06-18

**Authors:** Liliya Chamitava, Lucia Cazzoletti, Marcello Ferrari, Vanessa Garcia-Larsen, Aneza Jalil, Paolo Degan, Alessandro G. Fois, Elisabetta Zinellu, Sara S. Fois, Anna Maria Fratta Pasini, Morena Nicolis, Mario Olivieri, Angelo Corsico, Roberto Bono, Pietro Pirina, Maria Elisabetta Zanolin

**Affiliations:** 1 Unit of Epidemiology and Medical Statistics, Department of Diagnostics and Public Health, University of Verona, 37134 Verona, Italy; liliya.chamitava@univr.it (L.C.); lucia.cazzoletti@univr.it (L.C.); elisabetta.zanolin@univr.it (M.E.Z.); 2 Unit of Respiratory Medicine, Department of Medicine, University of Verona, 37134 Verona, Italy; marcello.ferrari@univr.it; 3 Department of International Health, The Johns Hopkins Bloomberg School of Public Health, Baltimore, MD 21205, USA; vgla@jhu.edu; 4 Pakistan Institute of Medical Sciences, Shaheed Zulfiqar Ali Bhutto Medical University, Islamabad 44000, Pakistan; anezarao@gmail.com; 5 U.O. Mutagenesi e Prevenzione Oncologica, Ospedale Policlinico San Martino, 16132 Genova, Italy; paolo.degan@hsanmartino.it; 6 Department of Medical, Surgical and Experimental Sciences, University of Sassari, 07100 Sassari, Italy; agfois@uniss.it (A.G.F.); sara.solveig.fois@gmail.com (S.S.F.); 7 Unit of Respiratory Diseases, University Hospital Sassari (AOU), 07100 Sassari, Italy; elisabetta.zinellu@aousassari.it; 8 Department of Medicine, Section of General Medicine and Atherothrombotic and Degenerative Diseases, University of Verona, 37134 Verona, Italy; annamaria.frattapasini@univr.it; 9 Unit of Hygiene and Preventive, Environmental and Occupational Medicine, Department of Diagnostics and Public Health, University of Verona, 37134 Verona, Italy; morena.nicolis@univr.it; 10 Unit of Occupational Medicine, Azienda Ospedaliero Universitaria di Verona, 37134 Verona, Italy; mario.olivieri@univr.it; 11 Division of Respiratory Diseases, ERCS, S. Matteo, Hospital University of Pavia, 27100 Pavia, Italy; angelo.corsico@unipv.it; 12 Department of Public Health and Pediatrics, University of Turin, 10126 Turin, Italy; roberto.bono@unito.it

**Keywords:** oxidative stress, inflammation, asthma, chronic bronchitis

## Abstract

Introduction: The global burden of chronic airway diseases represents an important public health concern. The role of oxidative stress and inflammation in the pathogenesis of these diseases is well known. The aim of this study is to evaluate the behavior of both inflammatory and oxidative stress biomarkers in patients with chronic bronchitis, current asthma and past asthma in the frame of a population-based study. Methods: For this purpose, data collected from the Gene Environment Interactions in Respiratory Diseases (GEIRD) Study, an Italian multicentre, multicase-control study, was evaluated. Cases and controls were identified through a two-stage screening process of individuals aged 20-65 years from the general population. Out of 16,569 subjects selected from the general population in the first stage of the survey, 2259 participated in the clinical evaluation. Oxidative stress biomarkers such as 8-oxo-7,8-dihydro-2′-deoxyguanosine (8-oxodG), 8-isoprostane and glutathione and inflammatory biomarkers such as Fractional Exhaled Nitric Oxide (FENO) and white blood cells were evaluated in 1878 subjects. Results: Current asthmatics presented higher levels of FENO (23.05 ppm), leucocytes (6770 n/µL), basophils (30.75 n/µL) and eosinophils (177.80 n/µL), while subjects with chronic bronchitis showed higher levels of GSH (0.29 mg/mL) and lymphocytes (2101.6 n/µL). The multivariable multinomial logistic regression confirmed high levels of leucocytes (RRR = 1.33), basophils (RRR = 1.48), eosinophils (RRR = 2.39), lymphocytes (RRR = 1.26) and FENO (RRR = 1.42) in subjects with current asthma. Subjects with past asthma had a statistically significant higher level of eosinophils (RRR = 1.78) with respect to controls. Subjects with chronic bronchitis were characterized by increased levels of eosinophils (RRR = 2.15), lymphocytes (RRR = 1.58), GSH (RRR = 2.23) and 8-isoprostane (RRR = 1.23). Conclusion: In our study, current asthmatics show a greater expression of the inflammatory profile compared to subjects who have had asthma in the past and chronic bronchitis. On the other hand, chronic bronchitis subjects showed a higher rate of expression of oxidative stress biomarkers compared to asthmatic subjects. In particular, inflammatory markers such as circulating inflammatory cells and FENO seem to be more specific for current asthma, while oxidative stress biomarkers such as glutathione and 8-isoprostane appear to be more specific and applicable to patients with chronic bronchitis.

## 1. Introduction

The global burden of chronic airway diseases is increasing as an effect of the aging of the world’s populations and represents a major public health concern. Among these diseases, asthma is a common condition characterized by chronic airway inflammation and hyperresponsiveness resulting in repeated attacks of wheezing and breathlessness [1]. The global prevalence of asthma in adults is estimated around 4.5%, which increases to 8.6% if we consider self-reported symptoms of asthma as well [2]. In Italy, asthma prevalence has been reported to be still on the rise as well as the prevalence of allergic rhinitis [3]. There is a strong association between asthma and allergic and non–allergic rhinitis and the two conditions often coexist [4].

Chronic bronchitis is defined by the presence of chronic cough and phlegm on most days for at least three months a year for two consecutive years. Its median prevalence is reported to be 2.6%, with wide variations across countries [5]. It affects about a third of patients with chronic obstructive pulmonary disease (COPD). Moreover, chronic bronchitis symptoms presence correlates with forced expiratory volume in 1 s (FEV_1_) decline and it is associated with worse health status in affected subjects [6].

It is described that inflammation and oxidative stress (OS) play an important role in the pathogenesis of obstructive airway diseases. Oxidative stress is an imbalance between production of reactive oxygen species (ROS) and antioxidant defensive capacity of the body [7,8]. The imbalance in oxidant–antioxidant system can be due to diminished antioxidant activity or increased production of ROS in human body [9].

Due to its anatomy, function and location, the lung is an organ easily affected by exogenous ROS [7,10], which could facilitate the onset of lung diseases and, as a consequence, the production of endogenous ROS [11]. Following inhalation of exogenous particles or inflammatory mediators, activated phagocytic cells accumulate in the lower respiratory tract leading to the production of a large amount of endogenous ROS [7] that are intended to protect the respiratory system against exogenous pollutants [11]. However, in chronic inflammatory conditions, macrophages and neutrophils become a persistent source of oxidative damage to the DNA and other cell components, which can cause lung disease [10]. Recently, it was shown that oxidative stress plays an important role in the pathogenesis of chronic bronchitis (CB) and asthma [12]. Oxidative stress caused by ROS can be estimated by either direct or indirect approaches by examining oxidation target products, including lipid peroxidation end products, protein and DNA oxidation end products. Being molecules in which the structure has been modified by ROS, they can be used to assess oxidative stress in humans. Other biomarkers of oxidative stress are antioxidant molecules, such as glutathione, protein thiols and enzymatic antioxidant activity. For this study, we have considered some of the most commonly studied biomarkers. In particular, 8-isoprostane, which is a product of lipid peroxidation and 8-oxo-7,8-dihydro-2′-deoxyguanosine (8-oxodG), a product of DNA oxidation. Among antioxidants we have examined glutathione that constitutes an important component of the antioxidant defense system and has been largely studied as a major oxidative stress biomarker [13]. Patients with CB have shown an increased OS with higher levels of malondialdehyde, a biomarker of lipid peroxidation and decreased levels of antioxidants, such as superoxide dismutase and glutathione, compared to a group of control subjects [12].

Inflammatory and immune cells in patients with asthma generate an increased number of ROS, leading to tissue injury and further oxidative stress [14,15]. Both endogenous and exogenous ROS shape the severity of asthma and COPD [16,17]. ROS and the products of their oxidative reactions enhance the inflammatory response in both asthma and COPD in different ways: through the impact on transduction mechanisms, activation of redox-sensitive transcriptions factors, and chromatin regulation, which leads to pro-inflammatory gene expression. Moreover, ROS diminish the activity of histone deacetylase-2 (HDAC-2) co-repressor, resulting in low efficacy of corticosteroids in COPD, severe asthma, and smoking asthmatics [17]. Oxidative stress also plays a role in allergic diseases such as rhinitis and atopic dermatitis [18]. House dust induces generation of H_2_O_2_ by nasal eosinophils in subjects with allergic rhinitis. In the blood of subjects with allergic skin conditions such as physical urticarias, reduced levels of antioxidants like vitamin E, catalase, and glutathione peroxidase have been found [18]. Several studies show that oxidative stress is associated with decreased lung function, i.e., lower values of FEV_1_ or forced vital capacity (FVC). An inverse correlation between FEV_1_ and a biomarker of lipid peroxidation (thiobarbituric acid-reactive substance—TBARS) has been found in a pilot group of non-smoking subjects from the general population [19,20]. The same authors found an inverse association between TBARS and FVC in subjects with COPD and asthma, and also reported that levels of various antioxidants (vitamins and beta-carotene) and the values of FEV_1_ and FVC are positively correlated [21]. A larger cross-sectional study confirmed this finding and furtherly described that TBARS and FVC are inversely associated in males but not in females, suggesting that gender might affect the relationship between oxidative stress and lung function [19].

In this work, we aimed to evaluate the association between oxidative stress biomarkers (8-oxodG, 8-isoprostane, glutathione) and inflammation (Fractional Exhaled Nitric Oxide-FENO, white blood cells) and respiratory diseases (current asthma, past asthma and chronic bronchitis).

## 2. Results

The main characteristics of the sample are presented in Table 1. The sample of current asthmatics presented higher levels of FENO (23.05 ppm), leucocytes (6770 n/µL), basophils (30.75 n/µL) and eosinophils (177.80 n/µL). Subjects with chronic bronchitis were more likely to be current smokers (53.8%) and had higher levels of GSH (0.29 mg/mL) and lymphocytes (2101.6 n/µL). The vast majority of never smokers and past smokers were asthmatics and controls, respectively. In regard to the non-hierarchical classification, the characteristics of the separate cases, i.e., current and past asthma vs. controls and chronic bronchitis vs. controls, are reported in the Supplementary Materials Tables S1 and S2.

The multivariable multinomial logistic regression confirmed the highest levels of leucocytes (RRR = 1.33, 95%CI(1.06–1.66)), basophils (RRR = 1.48, 95%CI(1.20–1.84)), eosinophils (RRR = 2.39, 95%CI (1.80–3.16)), lymphocytes (RRR = 1.26, 95%CI(1.03–1.55)) and FENO (RRR = 1.42, 95%CI(1.15–1.75)) in subjects with current asthma (Table 2). Subjects with past asthma had statistically higher level of eosinophils (RRR = 1.78, 95% CI (1.29–2.46)) as compared to controls; moreover, they showed an increased 8-isoprostane level (RRR = 1.17, 95%CI(0.97–1.40)) although not significant (Table 2). Subjects with chronic bronchitis were characterized by increased levels of eosinophils (RRR = 2.15, 95%CI (1.44–3.22)), lymphocytes (RRR = 1.58, 95%CI (1.07–2.34)) and GSH (RRR = 2.23, 95%CI (1.05–4.75)) (Table 2).

Most outcomes from Table 2 were confirmed in non-hierarchical models (Table 3) that considered each disease separately with respect to controls. As expected, the pattern of associations of biomarkers with current asthma and past asthma detected in non-hierarchical models and hierarchical models were very similar. Chronic bronchitis subjects had higher levels of eosinophils (RRR = 2.02, 95%CI(1.46–2.80)) and lymphocytes (RRR = 1.37, 95%CI(1.03–1.82)). In comparison with hierarchically classified cases, subjects with chronic bronchitis were characterized by increased levels of basophils (RRR = 1.56, 95%CI(1.17–2.10)) and 8-isoprostane (RRR = 1.23, 95%CI(1.02–1.49)) according to estimates of separate models for chronic bronchitis vs. controls.

## 3. Discussion

The main results derived from our study can be summarized as follows:

(1) Current asthmatics show, compared to subjects who have had asthma in the past and chronic bronchitis, a greater expression of the inflammatory profile in terms of FENO levels in the airways and an increase in leukocytes, basophils, lymphocytes and, most importantly, eosinophils counts in peripheral blood.

(2) Chronic bronchitis subjects, compared to current asthmatics and individuals who have suffered from asthma in the past, have a greater expression of oxidative stress biomarkers in terms of serum glutathione.

(3) Asthmatics who have suffered from asthma in the past have, compared to current asthmatics and chronic bronchitis, both a lower inflammatory profile in terms of FENO levels in the airways and circulating inflammatory cells, and a lower, but statistically not significant, expression of oxidative stress biomarkers.

Asthma is an inflammatory disease consisting of different endotypes with the involvement of several cells. Lymphocytes, in particular CD4+ Type-2 lymphocytes, play a crucial role in the pathogenesis of asthma [22]. Type 2 cytokines drive the recruitment of effector cells such as basophils and eosinophils. Once recruited, eosinophils participate in the modulation of the immune response, contributing to inflammation in asthma, especially airway epithelial cell damage, airway remodeling and hyperresponsiveness and mucus hypersecretion [23]. An overproduction of eosinophils is commonly found in many asthmatic patients, and it correlates with more severe disease [24]. Eosinophilic inflammation has also been reported in Chronic bronchitis and COPD [25]. Our results show that blood lymphocytes, basophils, and eosinophils are increased in current asthma compared to controls and these results are described both in the hierarchical model and in the non-hierarchical model.

Blood eosinophil count is an established biomarker in asthma. A high blood eosinophil count correlates with poor asthma control and with an increased risk of severe exacerbations and hospitalizations [26,27,28]. Another marker evaluated in our study is FENO. This is a validated and specific biomarker for T2-driven airway inflammation in asthma and its use in the clinical assessment of asthma is recommended by Guidelines from the American Thoracic Society [28]. In our study, FENO levels are significantly higher in current asthma compared to controls as we expected. Conversely, subjects that suffered from asthma in the past have an inflammatory profile, in terms of FENO and inflammatory blood cells, comparable to that of controls.

An increase in inflammatory cells, particularly eosinophils and lymphocytes, was also found in chronic bronchitis subjects in the hierarchical model. Increased airways inflammatory response could represent an early event in the pathological process of the disease or could be related to the presence of asthma comorbidity, therefore markers of inflammation could be useful in diagnosis and evaluation of prognosis and response to treatment. An increased blood eosinophil numbers might be indicative for a better therapeutic response to inhaled corticosteroids also in patients with COPD [29].

In this study, we have also assessed oxidative stress biomarkers, more specifically 8-oxodG, 8-isoprostane and glutathione. Many biomarkers of oxidative stress have been investigated in respiratory diseases [12,13,30]. An imbalance between oxidant markers (reactive oxygen species and their oxidation target products) and various antioxidants have been found in asthma and COPD [13,30]. A significant increase of serum glutathione in chronic bronchitis without airway obstruction compared to controls was found for the first time (hierarchical model). This is in contrast with the work of Nagaraj and Priyadarshini that found reduced levels of glutathione in whole blood of chronic bronchitis compared to controls [12]. We suppose that our findings could be explained by the fact that subjects were selected from the general population and were not in-hospital patients as in the study conducted by Nagaraj and Priyadarshini. This could also account for the relatively recent onset of CB in our subjects and the initial reaction of the organism to the increasing OS. As far as we know, there are no other case-control studies on glutathione levels in the blood of chronic bronchitis subjects. Many studies have evaluated GSH levels in COPD showing different results, and a reduction of GSH has been described in patients compared to controls [13], but few other studies have shown an increase in GSH levels. In particular, Ahmad et al. found an increase of total blood glutathione in 140 patients with COPD compared to 70 controls, and highlighted a negative correlation of this marker with FEV_1_%, [31]; Nadeem et al. also found higher levels of total blood glutathione in 82 patients with COPD compared to 22 healthy controls [32]. The discrepancies on blood glutathione in literature could be at least partially attributed to the analytical approach used to assay this marker and also to the fact that different studies are carried out in different populations, and there may be inter-individual variations especially in antioxidant capacity. Ochs-Balcom et al. found that glutathione levels were negatively associated with FEV_1_% in 218 subjects with chronic airflow limitations [21], therefore an increase of glutathione with worsening lung function can be assumed.

Our study shows that in non-hierarchical models, the difference in glutathione levels between subjects with chronic bronchitis and controls disappears. This could be due to the greater number of subjects with other comorbidities evaluated with this model. In the non-hierarchical model, we have found an increase in urinary 8-isoprostane in chronic bronchitis compared to controls and to asthmatic subjects. As for glutathione, literature lacks studies about 8-isoprostane in chronic bronchitis. In COPD, higher levels of 8-isoprostane have been described especially in exhaled breath condensate but also in urine. In particular, Praticò et al. described significantly higher levels of urinary 8-isoprostane in 38 patients with COPD compared to 30 healthy controls [33]; Santus et al. found an increase of urinary excretion for 8-isoprostane in COPD compared to healthy subjects for three consecutive days [34].

The most important strength of this study is that our analysis is based on a standardized protocol, which allowed a precise definition of each respiratory disease. Furthermore, the analysis highlights different chronic airway diseases with different pathogenesis and high epidemiological impact in the general population. Asthma and chronic bronchitis affect people in full working activity and therefore the socio-economic consequences are considerable. Having biomarkers that are easily detected, at low cost and useful in the diagnosis and treatment monitoring can be useful in clinical practice. As far as we know, there are no other studies that evaluate the biomarkers of inflammation and oxidative stress in subjects with current asthma in comparison with subjects with past asthma. These detections can be useful in avoiding unnecessary treatment and risks of overtreatment. Furthermore, although the biomarkers of oxidative stress have been extensively studied in COPD, the same cannot be said for their evaluation in patients with chronic bronchitis.

In conclusion, our results indicate that inflammatory markers such as circulating inflammatory cells and FENO seems to be more specific and useful for current asthma than for past asthma, while oxidative stress biomarkers such as glutathione and 8-isoprostane seem to be more specific and useful in patients with chronic bronchitis. More specifically, an increase in glutathione in subjects with chronic bronchitis (without airway obstruction) could be a physiological reaction counteracting the increment of oxidative stress in the initial phase of the disease. The assessment of non-invasive biomarkers in airway diseases is still an area of growing interest; both blood collection and FENO measurements are non-invasive tools, easy to collect and with high analytical reliability [35]. This work offers an evaluation of both inflammatory and oxidative stress biomarkers in chronic bronchitis, current asthma and past asthma. These biomarkers are certainly promising in the diagnosis and monitoring of disease progression.

## 4. Materials and Methods

### Study Design

The recruitment of potential subjects has been conducted within the GEIRD (The Genes Environment Interaction in Respiratory Diseases) survey, a multi-center, multi-case control study involving several Italian research centers [36]. Cases and controls were identified through a two-stage screening process by either picking from pre-existing surveys (ECRHS and ISAYA) [37,38] or by selecting new random samples of people from the general population aged 20-65 years.

In the first stage, the selected subjects received an email questionnaire concerning the presence of respiratory symptoms [3]. Over the following stage 2, all recipients reporting symptoms relating to asthma, chronic obstructive pulmonary disease (COPD) or chronic bronchitis (CB), plus a random group of people with rhinitis and no respiratory symptoms were invited to undergo a detailed clinical interview and lung function tests for accurate phenotyping, and to provide blood and urine samples. The GEIRD Project was approved by the Ethics Committee of the coordinating Center (Verona) and by the Ethics Committees of all other participating Centers. All centers gained written consent from each participant.

**Figure 1:**
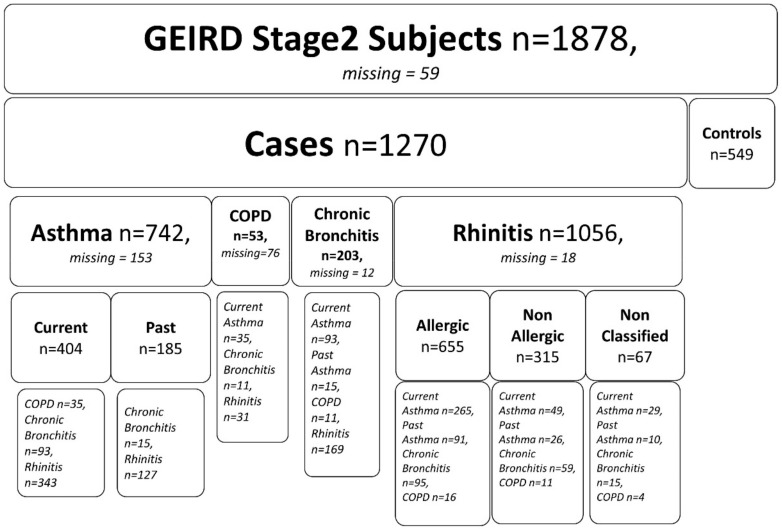
Number of participants in the Gene Environment Interactions in Respiratory Diseases (GEIRD) study of Verona, Pavia, Turin and Sassari.

In this work, data collected between years 2007 and 2013 in four Italian centers (Verona, Pavia, Turin and Sassari) has been analyzed. Out of 16,569 subjects selected in the first stage of GEIRD, 9741 (59%) completed the screening questionnaire. Of them, 4981 (51%) were furtherly selected to attend stage 2 and 2259 (45%) participated in the clinical survey. Data were available for 1878 subjects of the final group. Figure 1 shows the flowchart reflecting the overall situation of the GEIRD stage 2 study. It covers all respiratory pathologies including those not addressed in our analysis.

## 5. Identification of Cases and Controls

### 5.1. Cases Definition

Current Asthma definition: (1) self-reported asthma, plus one among (1.1) having had an asthma attack in the last 12 months, (1.2) current use of medications for asthma; or (2) asthma-like symptoms or use of medicines for breathing problems in the last 12 months, plus one among (2.1) a pre-bronchodilator FEV_1_/FVC <70% or <LLN with a positive reversibility test (i.e., FEV_1_ improvement >12% and >200 mL after administration of 400 μg of salbutamol), (2.2) a positive methacholine challenge test (PD20 < 1 mg).

Past asthma definition: The patients had a diagnosis and a history of asthma in the past but not current asthma, asthma-like symptoms, asthma attack in the last 12 months or current use of medication for asthma.

Chronic bronchitis (CB) definition: Subjects with self-reported cough and phlegm for most days over three consecutive months, over two consecutive years, with post-bronchodilator FEV_1_/FVC > 70% and > LLN.

Controls definition: Subjects who were not cases and had both (1) pre-bronchodilator FEV_1_/FVC>70% and > LLN; and (2) FEV_1_> 80% predicted; and (3) negative methacholine challenge test (PD20 < 1 mg); (4) negative skin prick tests.

The following diseases were also defined but were not considered as outcomes in our analysis:

COPD definition: Airflow limitation (post-bronchodilator FEV_1_/FVC <70% or <LLN).

Rhinitis definition: (1) Lifetime nasal allergies, including ‘hay fever’; (2) lifetime problem with sneezing, or a runny or blocked nose (without cold/flu); (3) recurrent nasal/eye symptoms in the presence of dust, pollen or animals. Atopic rhinitis subjects were positive to the skin prick test.

### 5.2. Study Subjects

In the current analysis, a total of 1230 subjects were hierarchically classified to build an outcome variable:

A total of 404 subjects had a diagnosis of current asthma. Among these, 93 also had chronic bronchitis, 35 had COPD, 265 had atopic rhinitis and 49 had non-atopic rhinitis.

A total of 185 subjects had a diagnosis of past asthma. Among these, 15 also had chronic bronchitis, 91 had atopic rhinitis and 26 had non-atopic rhinitis.

A total of 92 subjects had a diagnosis of chronic bronchitis and not one of asthma. Among these, 29 had atopic rhinitis and 35 had non-atopic rhinitis.

A total of 549 were controls, i.e., subjects who were not cases and presented with all of the following: (1) pre-bronchodilator FEV_1_/FVC>70% and >LLN; (2) FEV_1_> 80% predicted; (3) negative methacholine challenge test (PD20 < 1 mg); and (4) negative skin prick tests.

A non-hierarchical classification was also considered. The number of subjects in the asthma group did not change (404 subjects with current asthma and 185 subjects with past asthma). These subjects were characterized by the same number of coincident diseases, i.e., out of 404 cases with current asthma, 93 also had chronic bronchitis and 35 had COPD, 265 had atopic rhinitis and 49 had non-atopic rhinitis. Out of 185 cases with past asthma, 15 also had chronic bronchitis, 91 had atopic rhinitis and 26 had non-atopic rhinitis.

The number of cases with chronic bronchitis increased due to coincident cases of COPD and current/past asthma. Among the 273 subjects with chronic bronchitis, 93 also had current asthma, 15 had past asthma, 11 had COPD, 95 had atopic rhinitis and 59 had non-atopic rhinitis.

The number of controls in separate models did not change, i.e., it amounted to 549 subjects.

## 6. Clinical and Laboratory Measurements

### 6.1. Lung Function Testing

Forced expiratory volume in 1 s (FEV_1_) and forced vital capacity (FVC) were measured according to the American Thoracic Society reproducibility criteria [39]. Lung function values were expressed as a percentage of predicted values, and the lower limit of normal LLN for the FEV_1_/FVC was calculated according to Quanjer [40]. Spirometry was performed again 10 min after the administration of 400 μg salbutamol in subjects with FEV_1_/FVC <70% or <LLN. The subjects with FEV_1_/FVC >70% and >LLN underwent the methacholine challenge, according to a standardized protocol [41]. The test was considered positive if FEV_1_ decreased by 20% at a maximum cumulative dose <1 mg methacholine (PD20 < 1). The subjects were skin tested for a panel of 14 aeroallergens. The tested subject was considered to be atopic whenever positive to one or more of the tested allergens [42].

### 6.2. Urine Collection

The participants were asked to collect a spot quantity of the first morning urine in a clean container. Time of collection, number of smoked cigarettes and drugs taken right before collection were annotated (if a case) [3].

The container with the urine sample was stored at 4° for 24 h. Then, equal parts of 1 mL each were drawn and frozen at −80 °C pending further laboratory examination.

### 6.3. Biomarkers Evaluation

#### 6.3.1. 8-oxodG and the 8-isoprostane

The 8-oxodG (8-oxo-7,8-dihydro-2′-deoxyguanosine) and the 8-isoprostane (8-iso-prostaglandin F_2α_), both standardized by creatinine (ng/mg), were evaluated in our study with the immunosorbent assay kit ELISA (Cosmo Bio LTD, Tokyo Japan, and Cayman Chemical, Ann Arbor, MI, USA, respectively). In the assessment of creatinine concentration (mg/mL) another ELISA kit was used (Cayman Chemical, Ann Arbor, MI, USA).

#### 6.3.2. Glutathione (GSH)

Serum samples were directly collected into specially prepared tubes containing the preservative 2,6-di-tert-butyl-4-methylphenol (10 mmol/L) to reduce auto-oxidation and frozen at −80 °C. Samples were analyzed using high performance liquid chromatography with fluorescence detection of 7-fluorobenzo-2-oxa-1,3-diazol-4-sulfonic acid at excitation 385 nm and emission 515 nm [43].

#### 6.3.3. Fractional Exhaled Nitric Oxide (FENO)

FENO was measured at a flow rate of 0.05 L/second before the subjects underwent spirometry, in accordance with international guidelines (ATS/ERS 2005), using a chemiluminescence analyser (CLD88, Ecomedics, Switzerland). The same type of instrument was used in all centers, and was calibrated at 0 and 100 ppb as recommended by the manufacturer [44].

#### 6.3.4. White Blood Cell (WBC) Profiling (Verona Center Only)

The blood samples were collected and then preserved at −80 °C until their laboratory examination.

The hematology analyzer, the ADVIA 2120 (Siemens, Germany), was used to get WBC counts. The total WBCs were counted using the peroxidase method. The automated differential cell count was obtained using the peroxidase channel, providing relative proportions (percentages) of WBCs (basophils, eosinophils, neutrophils, monocytes, lymphocytes).

The absolute counts of basophils, eosinophils, neutrophils, monocytes and lymphocytes were obtained by multiplying the relative percentage by the total leukocyte count and then expressed in cell counts per microliter (n/μL).

### 6.4. Covariates

The following potential determinants of oxidative stress and inflammation were taken into consideration in current analyses: 8-oxodG, 8-isoprostane, GSH, FENO, WBC (basophils, eosinophils, neutrophils, leukocytes, monocytes, lymphocytes).

Age, gender, body mass index (BMI), study cohort [3,36,45], physical activity (≥4 times/week or ≥4 h/week, ≥1 time/week or ≥1 h/week, < 1 time/month or <1 h/week), smoking habit (never smoker, ex-smoker, current smoker), alcohol consumption (never, past, 5 g/day, 5–15 g/day, 15–30 g/day, 30–120 g/day), comorbidities: (1) heart attack, ictus, diabetes, (2) cancer were considered as potential confounders.

## 7. Statistical Analysis

The descriptive statistical analysis was used to define the main characteristics of the studied sample. The unconditional χ^2^ test, the Wilcoxon signed-rank test, the Kruskal–Wallis test and the t-test analyses were used for testing whether samples of cases and controls were different in terms of determinants and potential confounders age, gender and smoking status (α = 0.05).

Multinomial logistic regressions with relative-risk ratios (RRR) were used to estimate associations between hierarchically classified cases (dependent variable) and determinants (biomarker, independent variable). RRRs of all determinant covariates were expressed for one standard deviation (SD) increase. The biomarkers were inserted in each individual model separately, while all potential confounders were included in each regression model.

The cases were also analyzed considering a non-hierarchical classification that accounted for each disease separately. For this analysis, logistic regression and multinomial logistic regression were used in models for separate diseases: CB vs. controls and CA, PA vs. controls, respectively.

## Figures and Tables

**Table 1:** Characteristics of the sample, statistics for hierarchical classification of cases.

	Current Asthma (*n* = 404)	Past Asthma (*n* = 185)	Chronic Bronchitis (*n* = 92)	Controls (*n* = 549)	*p*-Value
	Median (IQR) or *n* (%)				
**Age**, years	44.09 (37.95–52.09)	44.35 (38.06–50.51)	46.91 (41.02–56.63)	49.38 (41.02–59.16)	**<0.001**
**Gender**, female	212 (52.48)	84 (45.41)	50 (54.35)	270 (49.18)	0.335
**Smoking habit**					<0.001
Never	192 (47.64)	99 (53.80)	39 (42.39)	288 (52.55)	
Past	111 (27.54)	53 (28.80)	17 (18.48)	166 (30.29)	
Current	100 (24.81)	32 (17.39)	36 (39.13)	94 (17.15)	
**FENO**, ppm	23.05 (12.0–45.0)	17 (9.6–30.0)	21 (10.6–34.0)	16.35 (11.5–24.7)	**<0.001**
**8-oxodG**, ng/mg*_creat_*	4.21 (2.17–7.90)	4.20 (1.88–7.96)	3.87 (1.90–7.19)	3.69 (1.89–7.46)	0.289
**8-isoprostane**, ng/mg*_creat_*	0.84 (0.39–1.55)	0.87 (0.26–1.68)	0.91 (0.48–1.49)	0.80 (0.29–1.73)	0.989
**GSH**, mg/ml	0.24 (0.18–0.28)	0.21 (0.16–0.27)	0.29 (0.22–0.34)	0.24 (0.19–0.29)	**0.014**
**Leucocytes**, cells/µL	6770 (5860–8220)	6540 (5900–7700)	6370 (5520–8290)	6350 (5395–7570)	**0.022**
**Basophils**, cells/µL	30.75 (20.10–44.17)	26.04 (17.70–36.12)	24.87 (19.40–43.80)	24.58 (15.88–34.35)	**0.001**
**Eosinophils**, cells/µL	177.80 (117.74–276.49)	156.87 (84.70–220.48)	164.92 (95.55–241.40)	111.25 (61.73–170.02)	**<0.001**
**Neutrophils**, cells/µL	4029.75 (3249.00–4995.92)	3987.50 (3250.08–4847.85)	3528.98 (32.46.96–4533.76)	3848.12 (3019.00–4748.41)	0.192
**Monocytes**, cells/µL	355.95 (289.38–441.98)	359.10 (287.50–431.49)	341.00 (280.28–421.20)	344.94 (283.14–428.42)	0.747
**Lymphocytes**, cells/µL	1968.68 (1663.20–2402.60)	1900.92 (1522.85–2363.60)	2101.60 (1562.00–2677.36)	1856.70 (1558.62–2167.30)	**0.017**

**Table 2:** Multivariable multinomial regression estimates of current asthma (CA), past asthma (PA) and chronic bronchitis (CB) in association with biomarkers.

	Hierarchically Classified Respiratory Diseases vs. Controls		
Biomarkers	CA	PA	CB
**Inflammatory Biomarkers, RRR(95%CI) per 1 SD increase**			
**FENO**	**1.42(1.15–1.75)**	1.24(0.97–1.59)	1.23(0.88–1.72)
**Leukocytes**	**1.33(1.06–1.66)**	1.14(0.86–1.51)	1.20(0.78–1.85)
**Basophils**	**1.48(1.19–1.84)**	1.12(0.85–1.49)	1.50(0.99–2.27)
**Eosinophils**	**2.39(1.80–3.16)**	**1.79(1.29–2.49)**	**2.15(1.44–3.22)**
**Neutrophils**	1.15(0.92–1.44)	1.05(0.80–1.39)	0.91(0.57–1.45)
**Monocytes**	1.05(0.85–1.29)	1.06(0.82–1.37)	1.07(0.71–1.59)
**Lymphocytes**	**1.26(1.03–1.55)**	1.13(0.88–1.46)	**1.58(1.07–2.34)**
**Oxidative Stress Biomarkers, RRR(95%CI) per 1 SD increase**			
**8-OxodG**	1.06(0.93–1.21)	1.11(0.95–1.30)	1.04(0.84–1.30)
**8-isoprostane**	1.07(0.91–1.26)	1.17(0.97–1.40)	1.16(0.93–1.46)
**Glutathione**	1.13(0.73–1.75)	0.65(0.30–1.42)	**2.23(1.05–4.75)**
Units of measure of cell biomarkers are (E+06/mL)/sd			

**Table 3:** Multivariable regression estimates of current asthma (CA), past asthma (PA) and chronic bronchitis (CB) in association with biomarkers. Non-hierarchical classification.

	Non-Hierarchically Classified (Separate) Respiratory Diseases vs. Controls		
Biomarkers	CA	PA	CB
**Inflammatory Biomarkers, RRR(95%CI) per 1 SD increase**			
**FENO**	**1.40(1.14–1.72)**	1.23(0.96–1.56)	1.33(1.02–1.75)
**Leukocytes**	**1.34(1.07–1.68)**	1.15(0.87–1.52)	1.13(0.82–1.56)
**Basophils**	**1.52(1.22–1.89)**	1.14(0.86–1.51)	**1.56(1.17–2.10)**
**Eosinophils**	**2.41(1.82–3.21)**	**1.80(1.30–2.50)**	**2.02(1.46–2.80)**
**Neutrophils**	1.16(0.93–1.44)	1.06(0.80–1.39)	0.88(0.63–1.40)
**Monocytes**	1.06(0.86–1.30)	1.06(0.82–1.38)	0.97(0.72–1.31)
**Lymphocytes**	**1.29(1.05–1.58)**	1.14(0.88–1.47)	**1.37(1.03–1.82)**
**Oxidative Stress Biomarkers, RRR(95%CI) per 1 SD increase**			
**8-OxodG**	1.05(0.92–1.21	1.11(0.94–1.30)	1.12(0.97–1.30)
**8-isoprostane**	1.07(0.90–1.28)	1.19(0.99–1.44)	**1.23(1.02–1.49)**
**Glutathione**	1.09(0.69–1.72	0.62(0.28–1.38)	1.25(0.77–2.03)
Units of measure of cell biomarkers are (E+06/mL)/sd Multinomial logistic regression and logistic regression were used in models for separate diseases: CA, PA vs. controls and CB vs. controls, respectively			

## Supplementary Materials

The following are available online at https://www.mdpi.com/1422-0067/21/12/4339/s1. Table S1. Characteristics of the sample (current and past asthma cases vs. controls). Non-hierarchical classification. Table S2. Characteristics of the sample (chronic bronchitis cases vs. controls). Non-hierarchical classification.
