# Supplementary material for: Biomarkers of Oxidative Stress and Inflammation in Chronic Airway Diseases
Source: Int J Mol Sci. 2020 Jun 18;21(12):4339. doi: 10.3390/ijms21124339 (PMC7353047; doi:10.3390/ijms21124339)
Supplement: Supplementary file 1 [file ijms-21-04339-s001.pdf]

Table S1. Characteristics of the sample (current and past asthma cases vs controls). Non-hierarchical classification.

	Current Asthma (n=404)	Past Asthma (n=185)	Controls (n=549)	p-value
	Median (IQR) or n (%)			
**Age**, years	44.09(37.95-52.09)	44.35(38.06-50.51)	49.38(41.02-59.16)	**<0.001**
**Gender**, female	212 (52.48)	84 (45.41)	270 (49.18)	0.263
**Smoking habit**				**0.048**
never	192 (47.64)	99 (53.80)	288 (52.55)	
past	111 (27.54)	53 (28.80)	166 (30.30)	
current	100 (24.81)	32 (17.39)	94 (17.15)	
**FENO**, ppm	23.05(12.00-45.00)	17 (9.6-30.0)	16.35(11.50-24.70)	**<0.001**
**8-oxodG**, ng/mg$_{creat}$	4.21 (2.17-7.90)	4.20(1.88-7.96)	3.69 (1.89-7.46)	0.178
**8-isoprostane**, ng/mg$_{creat}$	0.84(0.39-1.55)	0.87(0.26-1.68)	0.80(0.29- 1.73)	0.959
**GSH**, mg/ml	0.24 (0.18-0.28)	0.21 (0.16-0.27)	0.24 (0.19-0.29)	0.423
**Leucocytes**, n/µl	6770(5860-8220)	6540(5900-7700)	6350(5395-7570)	**0.008**
**Basophils**, n/µl	30.75(20.10-44.17)	26.04(17.70-36.12)	24.58(15.88-34.35)	**<0.001**
**Eosinophils**, n/µl	177.80(117.74-276.49)	156.87(84.70-220.48)	111.25(61.73-170.02)	**<0.001**
**Neutrophils**, n/µl	4029.75(3249.00-4995.92)	3987.50(3250.08-4847.85)	3848.12(3019.00-4748.41)	0.129
**Monocytes**, n/µl	355.95(289.38-441.98)	359.10(287.50-431.49)	344.94(283.14-428.42)	0.645
**Lymphocytes**, n/µl	1968.68(1663.20-2402.60)	1900.92(1522.85-2363.60)	1856.70(1558.62-2167.30)	**0.012**

Table S2. Characteristics of the sample (chronic bronchitis cases vs controls). Non-hierarchical classification.

	Chronic Bronchitis (n=203)	Controls (n=549)	p-value
	Median (IQR) or n (%)		
**Age**, years	45.42(39.10-54.68)	49.38(41.02-59.16)	**0.004**
**Gender**, female	97(47.78)	270(49.18)	0.734
**Smoking habit**			**<0.001**
never	90(44.33)	288(52.55)	
past	45(22.17)	166(30.30)	
current	68(33.50)	94(17.15)	
**FENO**, ppm	21.00(9.80-36.00)	16.35(11.50-24.70)	0.123
**8-oxodG**, ng/mg$_{creat}$	4.04(2.09-8.21)	3.69(1.89-7.46)	0.291
**8-isoprostane**, ng/mg$_{creat}$	0.96(0.45-1.59)	0.80(0.29-1.73)	0.330
**GSH**, mg/ml	0.25(0.20-0.31)	0.24(0.19-0.29)	0.073
**Leucocytes**, n/µl	6475(5520-7910)	6350(5395-7570)	0.275
**Basophils**, n/µl	29.95(20.34-44.10)	24.58(15.88-34.35)	**0.004**
**Eosinophils**, n/µl	171.30(118.08-261.03)	111.25(61.73-170.02)	**<0.001**
**Neutrophils**, n/µl	3576.31(3190.56-4533.76)	3848.12(3019.00-4748.41)	0.889
**Monocytes**, n/µl	327.91(277.76-414.50)	344.94(283.14-428.42)	0.518
**Lymphocytes**, n/µl	2110.74(1738.55-2391.32)	1856.70(1558.62-2167.30)	**0.005**
